# The Oncoprotein EVI1 and the DNA Methyltransferase Dnmt3 Co-Operate in Binding and *De Novo* Methylation of Target DNA

**DOI:** 10.1371/journal.pone.0020793

**Published:** 2011-06-10

**Authors:** Vitalyi Senyuk, Kavitha Premanand, Peng Xu, Zhijian Qian, Giuseppina Nucifora

**Affiliations:** Department of Medicine, University of Illinois at Chicago, Chicago, Illinois, United States of America; Tulane University Health Sciences Center, United States of America

## Abstract

*EVI1* has pleiotropic functions during murine embryogenesis and its targeted disruption leads to prenatal death by severely affecting the development of virtually all embryonic organs. However, its functions in adult tissues are still unclear. When inappropriately expressed, *EVI1* becomes one of the most aggressive oncogenes associated with human hematopoietic and solid cancers. The mechanisms by which EVI1 transforms normal cells are unknown, but we showed recently that EVI1 indirectly upregulates self-renewal and cell-cycling genes by inappropriate methylation of CpG dinucleotides in the regulatory regions of microRNA-124-3 (miR-124-3), leading to the repression of this small gene that controls normal differentiation and cell cycling of somatic cells. We used the regulatory regions of miR-124-3 as a read-out system to investigate how EVI1 induces *de novo* methylation of DNA. Here we show that EVI1 physically interacts with DNA methyltransferases 3a and 3b (Dnmt3a/b), which are the only *de novo* DNA methyltransferases identified to date in mouse and man, and that it forms an enzymatically active protein complex that induces *de novo* DNA methylation *in vitro*. This protein complex targets and binds to a precise region of miR-124-3 that is necessary for repression of a reporter gene by EVI1. Based on our findings, we propose that in cooperation with Dnmt3a/b EVI1 regulates the methylation of DNA as a sequence-specific mediator of *de novo* DNA methylation and that inappropriate EVI1 expression contributes to carcinogenesis through improper DNA methylation.

## Introduction

*Evi1* (Ecotropic virus integration 1) was identified as a common locus of retroviral integration in susceptible mice leading to the development of aggressive myeloid tumors [1]. The gene is highly conserved through evolution with homologs identified in eukaryotes from Xenopus to man [2]. In mouse and man the gene encodes a nuclear protein of 1051 amino acids with two domains of seven and three repeats of the zinc finger motif. In the mouse, homozygous disruption of *Evi1* leads to embryonic lethality (E10.5) with widespread hypocellularity and disruption of the developing organs [3], suggesting that this gene plays a critical role during organogenesis and morphogenesis as well as in cellular proliferation and differentiation. The role of this gene in adult tissues is less clear. Conditional deletion of *Evi1* in adult murine HSCs leads to a failure of their repopulating ability [4] whereas its forced expression in HSC upregulates cell division and self-renewal [5]. In patients, the inappropriate activation of *EVI1* is associated with development or progression of myeloid leukemia [6], [7] and solid cancers [8]–[11]. *In vitro* studies have shown that EVI1 blocks the TGF-beta [12] and the INF-alpha [13] pathways, and that interacts with many transcription factors, including GATA1 [14], RUNX1 [15], and PU.1 [16], presumably altering their functions. Finally, EVI1 has the ability to interact with co-repressors and co-activators of gene transcription [17].

DNA methylation, which occurs at the C5 position of a cytosine residue, is a major form of epigenetic modification with a role in gene silencing and genome stability [18]. Dense methylation of promoters causes strong transcriptional repression [19]. Abnormal DNA methylation, which often affects tumor suppressor genes, is one of the most consistent epigenetic changes seen in cancers [20]. There are three known catalytically active DNA methyltransferases (DMTs) two of which, 3a and 3b, are *de novo* DMTs (dnDMTs) [21]. The signals by which dnDMTs recognize and target specific DNA sequences to be methylated are unknown.

Recently, we showed that EVI1 downregulates microRNA-124 (miR-124), a group of small genes that control differentiation and cell cycling of normal hematopoietic cells [22]. We further reported that the downregulation occurs through EVI1-induced methylation of CpG dinucleotides located upstream of miR-124-3. This *de novo* methylation leads to miR-124 repression and to the upregulation of genes required for self-renewal and cell division such as *Bmi1* and *Cyclin D3* that are regulated by miR-124 [22]. Here, we show that EVI1 physically interacts with dnDMTs and that the two proteins form an enzymatically active complex that cooperatively binds to specific regulatory regions of miR-124-3. The proteins cooperate in repressing a reporter gene stably integrated in a cell line and are capable of DNA methylation *in vitro*. Based on our findings, we propose that EVI1 participates in DNA methylation as a novel sequence-specific co-factor of *de novo* DNA methylation and that, when inappropriately expressed, alters the differentiation status of a cell by improper methylation of genes, ultimately leading to cell transformation.

## Results

### EVI1 requires the cooperation of Dnmt3b to efficiently repress the regulatory region of miR-124-3

**Figure 1:**
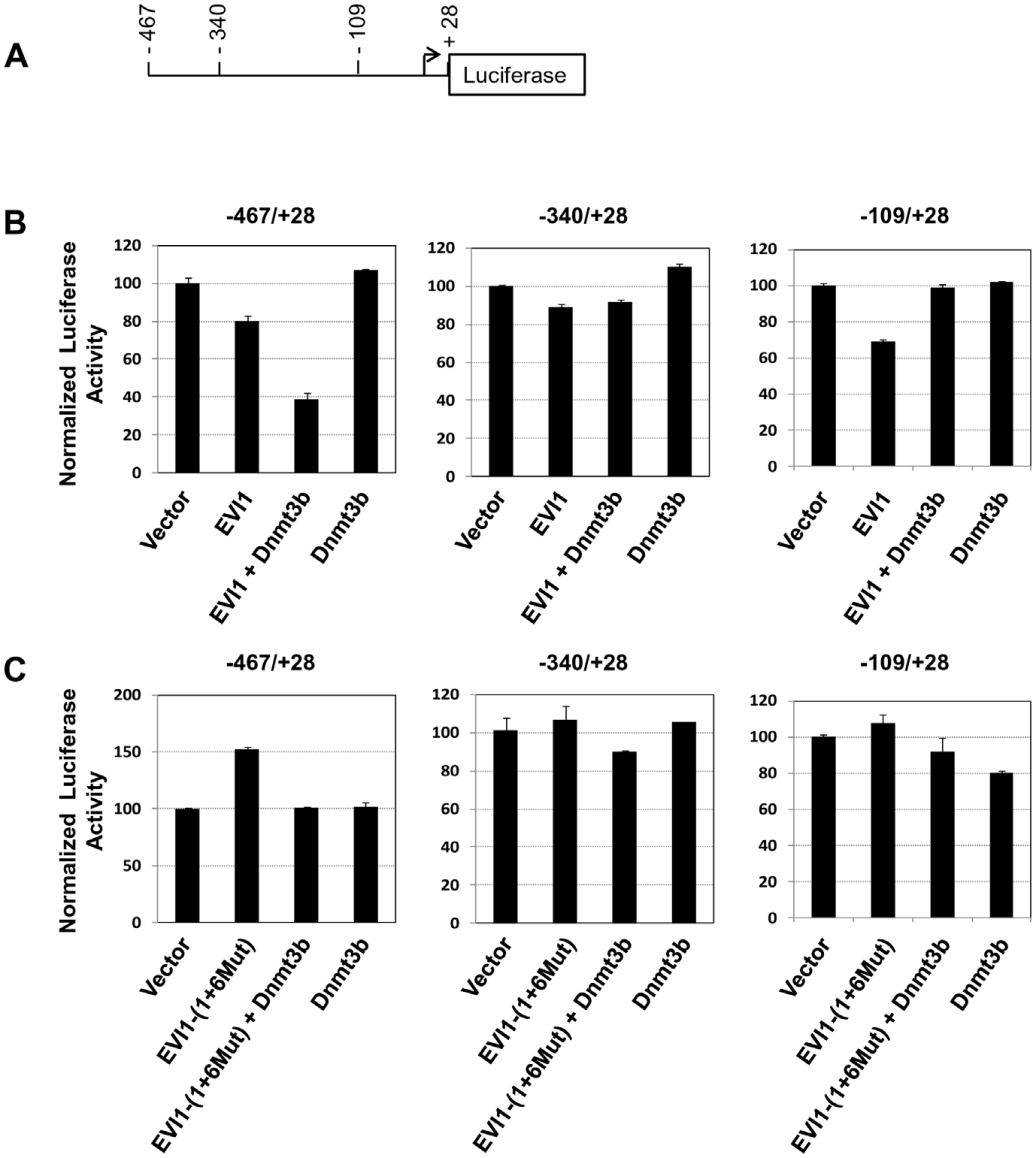
EVI1 synergizes with Dnmt3b to repress the regulatory regions of miR-124-3. A. Diagram of miR-124-3-Luciferase reporter gene. Numbers indicate the nucleotide boundaries of the reporter constructs and they are numbered respect to the stem-loop start in the miR-124-3 gene taken as +1 and indicated by the arrow. B, C. NIH-3T3 cells stably transfected with reporter plasmids (−467/+28 or −340/+28 or −109/+28) were transiently co-transfected with effector plasmids as shown in the panels. Cell extracts were used for quantification of the reporter gene as described in Material and Methods.

We previously reported that the expression of EVI1 in murine HSC induces the upregulation of cell division and an enhancement of self-renewal as a result of miR-124 silencing through DNA methylation of miR-124 regulatory regions. MiR-124 regulates these pathways [23]. We also reported that the EVI1 mutant EVI1-(1+6Mut), which contains two point mutations in two zinc finger motifs [14], [22], does not significantly alter these pathways and is unable to significantly methylate the regulatory regions of miR-124-3 [22]. To evaluate the mechanism by which EVI1 can induce methylation of DNA, we inserted the regulatory regions of miR-124-3 between nucleotides −467 and +28 upstream of the Luciferase reporter gene and used this plasmid as a read-out system. We also generated two additional reporter constructs that contained the region between nucleotides −340 and +28 and between nucleotides −109 and +28 (Figure 1A). To avoid artifacts due to transient transfection of the reporter gene and to provide a chromatin structure for the reporter gene, these plasmids were stably integrated into NIH-3T3 cells. Multiclonal populations of stably transfected NIH-3T3 cells were used to read the response of the artificial promoter to effector plasmids (empty vector, EVI1, EVI1-(1+6Mut), Dnmt3b, and EVI1+Dnmt3b). The results of the reporter gene assays are shown in Figures 1B and 1C. Firstly, we noted that when EVI1 was expressed by itself, it was able to repress moderately (10% to 15%) the three artificial promoters (Figure 1B). In contrast, when the mutant EVI1-(1+6Mut) was expressed there was a positive response of the reporter gene (Figure 1C). Because, as we will show later, this mutant does not significantly bind to the artificial promoter or interact with Dnmt3b, we believe that this upregulation could be caused by effects similar to squelching and was not investigated further. Secondly, we noted that Dnmt3b was unable to significantly repress the promoter by itself (Figure 1B and 1C). Finally we observed that there was a significant cooperative repressive effect between EVI1 and Dnmt3b on the response of the reporter. However, this increased repression was observed only when the artificial promoter contained the −467/−340 region (compare the third bars in the three panels of Figure 1B), suggesting that this short fragment is required for cooperation between EVI1 and Dnmt3b. No cooperative repression was observed when EVI1-(1+6Mut) was expressed with Dnmt3b (Figure 1C).

### EVI1 interacts with Dnmt3a *in vivo*

**Figure 2:**
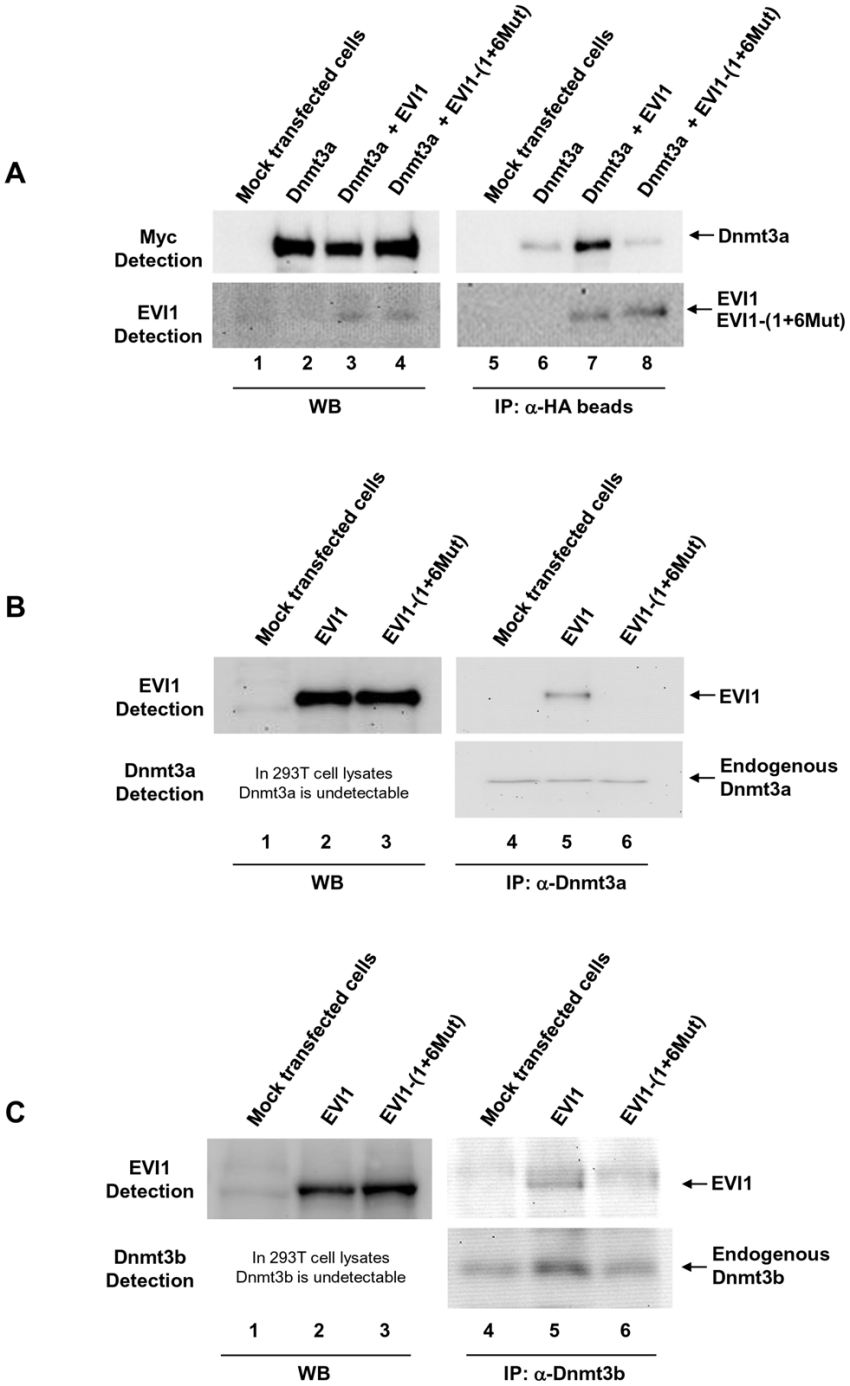
EVI1 interacts with *de novo* DNA methyltransferases. A. EVI1 (lane 7) but not EVI1-(1+6Mut) (lane 8) interacts with Dnmt3a. 293T cells were co-transfected with Myc-tagged Dnmt3a alone (lanes 2 and 6) or in combination with HA-tagged EVI1 or EVI1-(1+6Mut) (lanes 3 and 7, or lanes 4 and 8, respectively). Two days after transfection cell lysates were collected and incubated with anti-HA beads (lanes 5 to 8) followed by IP. The immunoprecipitated proteins (lanes 5 to 8) and proteins from the cell lysates (lanes 1 to 4) were separated by electrophoresis, transferred to a PVDF membrane and probed as marked in the Figure. Lanes 1 and 5 represent the results with mock transfected cells. B, C. 293T cells were transfected with a plasmid encoding EVI1 (lanes 2 and 5) or EVI1-(1+6Mut) (lanes 3 and 6). Lanes 1 and 4 represent mock-transfected cells. Two days after transfection cell lysates were collected and incubated with anti-Dnmt3a (B) or anti-Dnmt3b (C) antibody (lanes 4 to 6) followed by IP. The immunoprecipitated proteins (lanes 4 to 6) and proteins from cell lysates (lanes 1 to 3) were separated by electrophoresis, transferred to a PVDF membrane and probed as marked in the Figure.

It is generally accepted that proteins that cooperate in promoter regulation often interact between themselves at the promoter site. Therefore, to determine whether an interaction exists between EVI1 and DNA methyltransferases, we used first transient transfection of cell lines and co-IP assays with the wild type EVI1 and the mutant EVI1 as a negative control. Exponentially growing 293T cells were co-transfected with Dnmt3a alone or in combination with either EVI1 or EVI1-(1+6Mut). Two days after transfection, EVI1 and EVI1-(1+6Mut) were immunoprecipitated with anti-HA antibody, which recognizes HA-tagged EVI1 and EVI1-(1+6Mut), and the immunoprecipitated proteins were separated by electrophoresis and analyzed by Western blot. As shown in Figure 2A, Dnmt3a was co-precipitated by anti-HA antibody in extracts of cells co-transfected with EVI1 (lane 7, top) indicating that Dnmt3a interacts with EVI1 *in vivo*. In contrast, in the absence of EVI1 (lane 6, top) or in the presence of EVI1 mutant (lane 8, top), the antibody was not able to co-precipitate Dnmt3a significantly.

To confirm the results, we evaluated the interaction of EVI1 with endogenous dnDMTs using antibodies specific to Dnmt3a and Dnmt3b. 293T cells were transfected with EVI1 and the assays were performed as described above. As shown in Figures 2B and 2C, EVI1 co-precipitates with endogenous Dnmt3a (Figure 2B, lane 5) and Dnmt3b (Figure 2C, lane 5), suggesting that EVI1 forms complexes with dnDMTs *in vivo*. In contrast, EVI1-(1+6Mut), which does not induce miR-124-3 methylation *in vivo* [22], did not bind to the dnDMTs *in vivo* (lane 6 in Figures 2B and 2C). The interaction of endogenous EVI1 and Dnmt3a/Dnmt3b was confirmed with endogenous proteins in K562 cells (data not shown).

### Zinc finger motifs 1 and 6 of EVI1 are required for interaction with the catalytic domain of Dnmt3a

**Figure 3:**
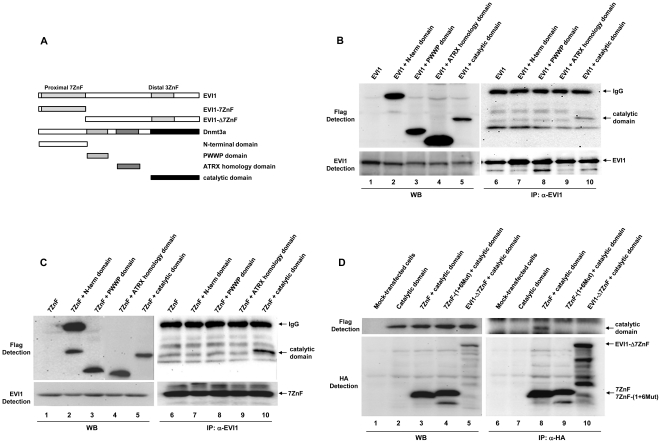
The zinc finger motifs 1 and 6 of EVI1 are required for interaction with the catalytic domain of *de novo* DNA methyltransferases. A. Schematic diagram of EVI1 and Dnmt3a shows the relevant domains analyzed in this study. B. EVI1 interacts with the catalytic domain of Dnmt3a. 293T cells were transiently co-transfected with full-length EVI1 and each one of the Flag-tagged separate domains of Dnmt3a as indicated, and analyzed as described in Figure 2. Lanes 1 to 5 show the expression of EVI1 (bottom panel) and Dnmt3a domains (top panel) in the transfected cells. Lanes 6 to 10 show the proteins after IP with anti-EVI1 antibody. C. The proximal zinc finger domain of EVI1 interacts with the catalytic domain of Dnmt3a. 293T cells were transiently co-transfected with the HA-tagged EVI1 proximal domain (7ZnF) and each one of the Flag-tagged domains of Dnmt3a. The cells were processed and analyzed as described above. Lanes 1 to 5 show the expression of 7ZnF domain (bottom panel) and Dnmt3a domains (top panel) in the transfected cells. Lanes 6 to 10 show the proteins after IP with anti-EVI1 antibody. The proximal domain, 7ZnF, interacts only with Dnmt3a catalytic domain (lane 10). D. Zinc finger motifs 1 and 6 must be intact for interaction with Dnmt3a. 293T cells were transiently co-transfected with the Flag-tagged catalytic domain of Dnmt3a alone (lanes 2 and 7) or in combination with the HA-tagged 7ZnF domain (lanes 3 and 8) or with the mutant 7ZnF-(1+6Mut) domain (lanes 4 and 9) or with EVI1-Δ7ZnF (lanes 5 and 10). The proteins in the cell extracts were analyzed by Western blot after co-IP with anti-HA antibody. Interaction is observed only when the intact proximal domain is expressed (lane 8).

EVI1 is a complex protein that contains ten zinc finger motifs, seven of which are closely grouped together at the N-terminus (proximal zinc finger domain) and the remaining three motifs are located at the C-terminus (distal zinc finger domain) (Figure 3A). We earlier showed that the proximal zinc finger domain is crucial for EVI1-induced methylation of DNA, which is not observed when this domain is mutated in EVI1-(1+6Mut) [22]. Dnmt3a is also a complex protein with several functional domains that have been characterized (Figure 3A). To map the interaction between the two proteins, we first tested full length EVI1 with each one of the Dnmt3a domains. The analysis was carried out by transient transfection of 293T cells and co-IP assays as described above. The results indicate that the full length EVI1 preferentially interacts with the C-terminus of Dnmt3a, which contains the catalytic domain (Figure 3B, lane 10). Because the results shown in Figure 2 suggested that the proximal zinc finger domain of EVI1 could be involved in the interaction, we tested the ability of this domain to interact with Dnmt3a. We transfected 293T cells with the proximal domain alone or in combination with domains of Dnmt3a. The results of the Western blot analyses confirm that the proximal domain of EVI1 interacts with the catalytic region of Dnmt3a (Figure 3C, lane 10) and that mutation of zinc finger motifs 1 and 6 abrogates the interaction (Figure 3D, compare top lanes 8 and 9). Finally, to exclude the role of any other EVI1 regions in the interaction, we co-expressed an EVI1 mutant that lacks the proximal zinc finger domain (EVI1-Δ7ZnF) with the catalytic domain of Dnmt3a. The results confirm that the interaction occurs through the proximal zinc finger domain and that no other region of EVI1 significantly contributes to the association between the two proteins (Figure 3D, compare top lanes 8 and 10).

### EVI1 and dnDMTs co-immunoprecipitate with the genomic regulatory region of miR-124-3

**Figure 4:**
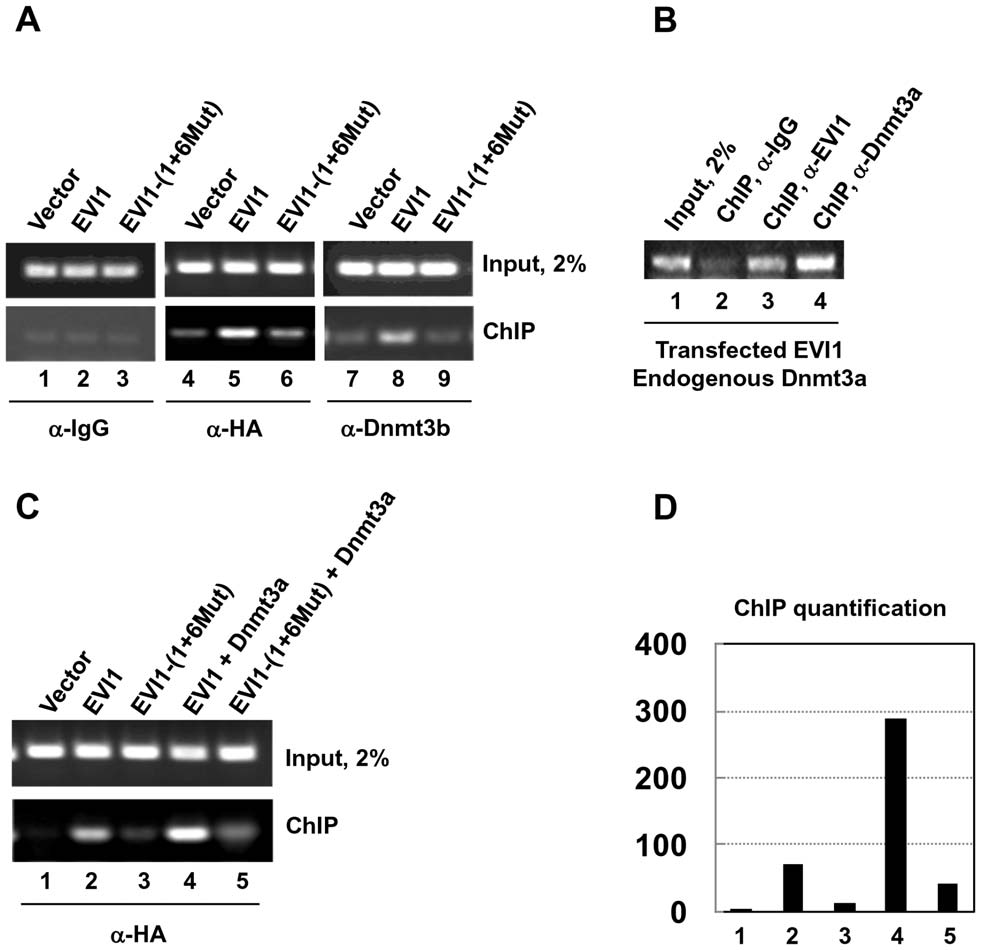
EVI1 and *de novo* DNA methyltransferases occupy the regulatory region of miR-124-3. A. ChIP assay was performed with chromatin fragments obtained from 293T cells transiently transfected with empty vector (lanes 1, 4, and 7), EVI1 (lanes 2, 5, and 8) and EVI1-(1+6Mut) (lanes 3, 6, and 9). For the ChIP, we used IgG (lanes 1 to 3) or anti-HA (lanes 4 to 6) or anti-Dnmt3b (lanes 7 to 9) antibodies. Transfected EVI1 (lane 5, lower panel) and endogenous Dnmt3b (lane 8, lower panel) are present together on the putative miR-124-3 promoter. In contrast, when EVI1 is not expressed (lanes 4 and 7) or is mutated (lanes 6 and 9) Dnmt3b is less capable of binding to chromatin. Lanes 1–3 represent negative control with unspecific IgG. B. Dnmt3a is also enriched at the putative miR-124-3 promoter in EVI1-transfected cells (lane 4). ChIP assay was performed with chromatin fragments derived from 293T cells transiently transfected with EVI1. C. Cooperation between EVI1 and Dnmt3a in promoter occupancy. 293T cells were transiently transfected with the empty vector (lane 1), EVI1 and EVI1-(1+6Mut) alone (lanes 2 and 3) or in combination with Dnmt3a (lanes 4 and 5). The cells were used for chromatin fragments isolation/ChIP with anti-HA antibody. Normal EVI1 and Dnmt3a are more efficient in ChIP (compare lanes 2 and 4). D. ChIP quantification. The signal for cells transfected with the empty vector was arbitrarily taken as 1. Lanes numbering in C and D is the same.

We used ChIP assays to determine whether EVI1 and the Dnmt3 proteins associate with the regulatory region of miR-124-3. 293T cells were transiently transfected with plasmids encoding EVI1 or mutant EVI1-(1+6Mut) and processed as indicated in Materials and Methods. The nuclear proteins associated with chromatin were immunoprecipitated with HA antibody, which recognizes the epitope-tagged EVI1 and mutant EVI1-(1+6Mut), or with Dnmt3b antibody. As shown in Figure 4A, lanes 5 and 8, an antibody to EVI1 or Dnmt3b efficiently co-precipitates a DNA fragment from the regulatory region of miR-124-3 when EVI1 is expressed, suggesting that both proteins associate with this DNA region. In contrast, when EVI1 is absent or mutated, the Dnmt3b antibody is unable to precipitate well the same fragment, supporting the notion that EVI1 could have a role in directing and efficiently binding the DNA methyltransferase to this DNA. Similar ChIP results were obtained with antibody to Dnmt3a in 293T cells transfected with EVI1. Not surprising, given the homology between the two DNA methyltransferases, Dnmt3a associates with this DNA region when EVI1 is expressed (Figure 4B). To confirm the qualitative ChIP results and to quantify the chromatin immunoprecipitated by EVI1 or by EVI1 mutant in presence or absence of added Dnmt3a, we transiently transfected 293T cells with EVI1 or EVI1-(1+6Mut) alone or in combination with Dnmt3a, followed by ChIP assays and used PCR for ChIP quantification. The results (Figure 4, C and D) confirm the previous qualitative ChIP results and also show that Dnmt3a significantly increases the interaction/strength between EVI1 and chromatin (compare lanes 2 and 4 in Figures 4C and 4D). This effect is strongly reduced or not observed at all when the EVI1 mutant is used. It is possible that EVI1-(1+6Mut) retains a minimal affinity with the miR-124-3 regulatory elements either directly or through unknown factor(s).

### An EVI1-Dnmt3b complex interacts with a specific miR-124-3 regulatory element

**Figure 5:**
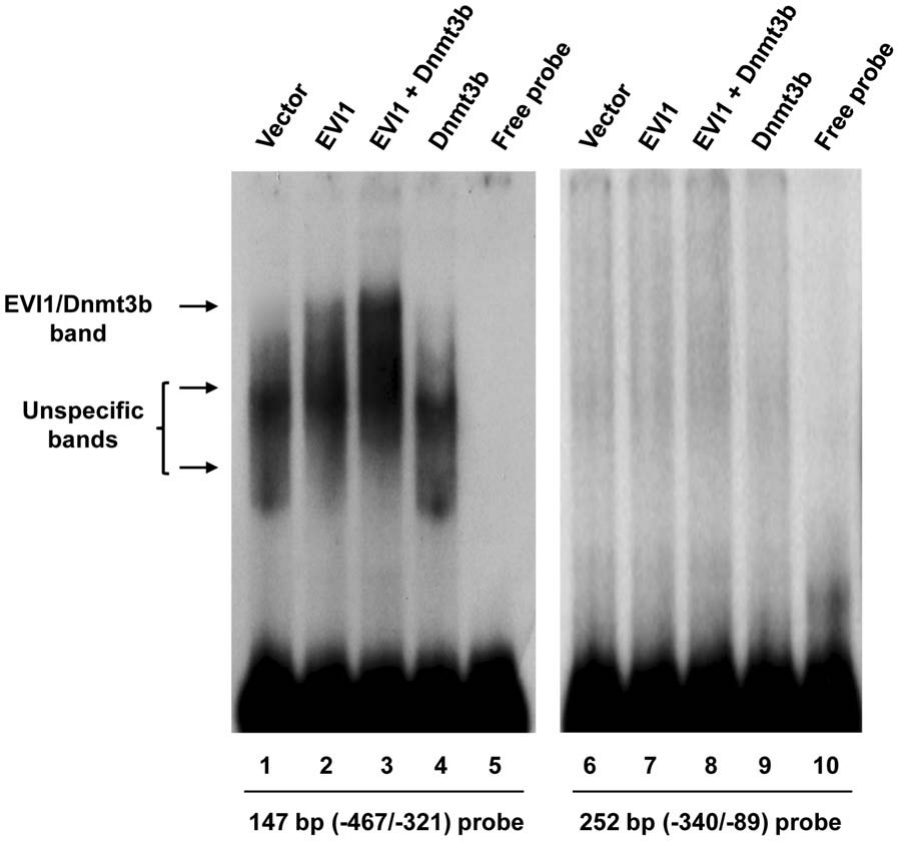
EVI1-Dnmt3b interact with the putative miR-124-3 promoter. 293T cells were transiently transfected with the empty vector (lanes 1 and 6), EVI1 (lanes 2 and 7), EVI1 and Dnmt3b (lanes 3 and 8) and Dnmt3b (lanes 4 and 9) and used for nuclear lysates isolation/EMSA assay. Two end-labeled probes of 147 bp (left panel) and 252 bp (right panel) were used. No significant signal is observed with the longer 252 bp probe (right panel). In contrast, a strong unspecific background is observed with the 147 bp probe (lane 1) or when exogenous Dnmt3b is expressed (lane 4). A slower migrating band appears with EVI1 (lane 2) and is enhanced by the expression of exogenous Dnmt3b (lane 3).

The results we have shown so far suggest first, that EVI1 and Dnmt3b cooperate in the repression of the regulatory elements of miR-124-3; second, that the DNA region between nucleotides −467 and −340 is necessary for repression; and third, that these two proteins could form a complex with DNA regulatory element(s) of miR-124-3. We used EMSA to confirm that the proteins associate with miR-124-3 elements. We used two probes: the 147 bp segment (between nucleotides −467 to −321) that is important for repression (Figure 1B), and a 252 bp segment between nucleotides −340 to −89. The probes were end-labeled PCR fragments amplified from genomic DNA and the nuclear extracts were obtained from transfected 293T cells as described in Materials and Methods. The results, shown in Figure 5, indicate that EVI1 and Dnmt3b, alone or co-expressed in the cells, fail to interact with the longer 252 bp probe (right panel). However, the results are quite different when we used the probe corresponding to the DNA fragment important for repression (left panel) in that they clearly indicate that the 147 bp probe interacts with proteins from the nuclear extracts. Despite a high background of diffuse unspecific bands (lane 1), EVI1 alone (lane 2) and especially in presence of added Dnmt3b (lane 3) has a clear affinity for the 147 bp miR-124-3 probe. Moreover, the addition of excess (50×) cold 147 bp competitor disrupted the complex between EVI1 and 147 bp probe whereas 50× excess of 252 bp competitor had very limited effect on the EVI1 and 147 bp probe interaction (data not shown). Several DNA sequences have been reported as potential DNA binding site of the proximal zinc finger domain of EVI1 [24], [25]. However, we did not find any significant homology between the published consensus and the 147 bp that form the DNA probe to which EVI1/dnDMT bind (data not shown).

### Dnmt3b and EVI1 form an enzymatically active complex that methylates DNA *in vitro*

**Figure 6:**
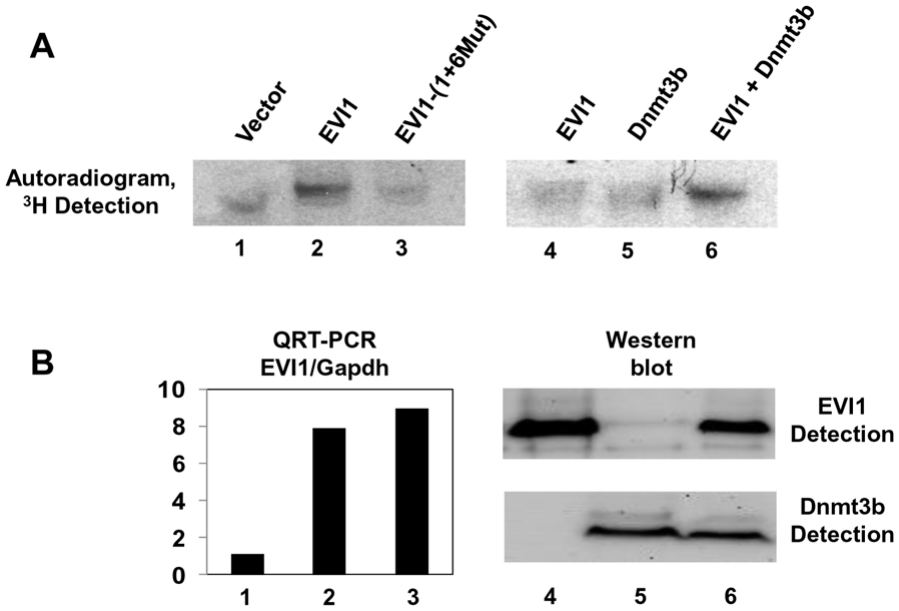
EVI1 and *de novo* DNA methyltransferases form an enzymatically active complex. A. ES cells stably transfected with the empty vector (lane 1), EVI1 (lane 2) or EVI1-(1+6Mut) (lane 3) were used for *in vitro* DMTase assay as described in Material and Methods. 293T cells were transiently transfected with plasmids encoding EVI1 (lane 4), Dnmt3b (lane 5), or EVI1 and Dnmt3b (lane 6), and the IP proteins were used for *in vitro* DMTase assay. A strong band evident in lane 2 represents the DNA methyltransferase activity of EVI1 and endogenous dnDMTs from ES cells. A strong band evident in lane 6 results from the activity of co-transfected Dnmt3b and EVI1. B. Quantitative RT-PCR shows that the expression of EVI1 (lane 2) and EVI1-(1+6Mut) (lane 3) in ES cells is comparable. Western blot analysis shows the expression of EVI1 (lane 4), Dnmt3b (lane 5) and EVI1+Dnmt3b (lane 6) in 293T cells used for *in vitro* DMTase assay.

We previously reported that EVI1 induces DNA methylation *in vivo* leading to miR-124 repression [22] and here we have shown that EVI1 and Dnmt3b form a complex that interacts with a regulatory region of miR-124-3 required for repression of a stably integrated reporter gene. To determine whether this complex possesses methyltransferase activity we used an *in vitro* DNA methyltransferase assay. Because it was reported that murine ES cells have relatively high expression of DNA methyltransferases [26], we stably transfected ES cells with an empty vector or with a vector encoding EVI1 or EVI1-(1+6Mut). Proteins in ES cell lysates were immunoprecipitated with α-HA antibody that recognizes the epitope-tagged EVI1 and EVI1-mutant. After several washes, the anti-HA beads with the adsorbed proteins were incubated with the target/substrate DNA (879 bp PCR fragment containing the regulatory region of miR-124-3) in the presence of ^3^H-Ado-Met, and the reaction mixture was processed as described in Material and Methods. As shown in Figure 6A, there is no significant ^3^H-methylation of the substrate when we used the proteins immunoprecipitated from ES cells expressing the empty vector (lane 1) or the vector encoding the mutant EVI1 (lane 3). In contrast, when the proteins were immunoprecipitated from EVI1-expressing ES cells, a strong band was detected (lane 2). We repeated this assay with 293T cells transiently transfected with EVI1 (lane 4), Dnmt3b (lane 5), or EVI1 and Dnmt3b (lane 6). With these cells we observed a fainter but clear ^3^H-methylation band when EVI1 (lane 4) or Dnmt3b (lane 5) were expressed alone. The ^3^H-methylation band became much stronger when the two proteins were co-expressed in the cells (lane 6), supporting the data we obtained with ChIP and EMSA suggesting that the proteins co-operate in binding to the DNA and in *de novo* DNA methylation. Figure 6B shows the expression of EVI1 or EVI1-(1+6Mut) in ES cells obtained by quantitative PCR (left panel) and the expression of EVI1 and Dnmt3b in 293T cells obtained by Western blot analysis (right panel).

## Discussion

Since the identification of EVI1 over 20 years ago [1], the inappropriate expression of this gene has been associated with very aggressive hematopoietic cancers and especially with myelodysplastic syndromes (MDS) of patients in whom *EVI1* is often activated following a chromosomal rearrangement at chromosome 3 band q26 [27]. Several publications on the role of EVI1 in hematopoietic diseases showed that this protein not only affects factors that control normal hematopoiesis such as RUNX1, GATA1, and PU.1 [14]–[16], but also deregulates two critical signaling networks controlled by TGF-beta and IFN-alpha [12], that influence cell growth and differentiation in the bone marrow. Therefore, it was thought that the major role of EVI1 in hematopoietic transformation was based on inappropriate interactions with factors and cytokines that monitor cell growth and differentiation in a way similar to that proposed for leukemia-associated and hematopoiesis-specific factors such as RUNX1 or GATA1 or PU.1 [28]–[30]. In the last few years, however, it was clearly shown that EVI1 is also activated in several aggressive solid cancers such as lung, colon, and breast cancers [8]–[11], which occur in tissues normally controlled by alternative sets of differentiation factors. These new reports raise the question of the role of EVI1 in a normal cell versus a neoplastic cell in which the gene is inappropriately expressed irrespective of the tissue of origin. Very little is known about the normal role of EVI1 in a cell. Perhaps the earliest clue of a potential normal function of EVI1 can be found in the description of murine embryos in which *Evi1* had been targeted and disrupted by homologous recombination [3]. In contrast to the phenotype of RUNX1−/− (or GATA1−/−, or PU.1−/−) embryos, which showed precise and well defined abnormalities mostly confined to developing hematopoietic compartments [31]–[33], the defects induced by *Evi1* disruption were not only extended to virtually all the developing organs but the disruption of *Evi1* had also an impact on the overall cellularity of the developing embryo [3], suggesting a pleiotropic, rather than a narrowly tissue-defined, role of this protein that was essential during embryogenesis. We believe that a second clue about a potential function of EVI1 was found in multiple publications linking EVI1 to MDS and in the observation that MDS cells are characterized by overall inappropriate DNA methylation.

We and others recently reported that indeed a link exists between EVI1 and DNA methylation [22], [34] and here we used our previous finding as a basis to understand the molecular mechanism by which EVI1 is capable of DNA modification. We have specifically looked at the potential co-operation of EVI1 with dnDMTs because dnDMTs are involved in *de novo* methylation of DNA. The biochemical characteristics of dnDMTs have been extensively studied during the last few years. In addition to the dissection of the dnDMTs major functional domains [21], it was reported that the proteins require the catalytically inactive isoform Dnmt3L for maximum activity and substrate affinity [35] and for periodic DNA methylation [36], that specific lysine methylation in proteins such as histones leads to preferential DNA methylation of these genes [37], and that active DNA demethylation is crucial for epigenetic reprogramming and induced pluripotency [38], [39]. Several potential mechanisms have been proposed for DNA methylation by dnDMTs including their interaction with unknown factors, selective anchoring of dnDMTs on methylated nucleosomes [40], cooperation with lysine demethylases [41] or methyltransferases [42], [43], interaction with centromere proteins [44], and association with sequence-specific DNA binding proteins [45]–[47]. Interestingly, though, there is limited information available on the identity of factors that allow recognition of target DNA and on the precise mechanisms or the nature of the signals required for specific CpG methylation. The results that we present in this report using the regulatory region of miR-124-3 as a read-out system suggest that EVI1 forms a complex with dnDMTs that binds to specific regions of DNA and that is capable of CpG methylation. Our data also suggest that these two events, recognition of target DNA and CpG methylation by dnDMTs, require EVI1 for optimal efficiency. Therefore, these results also suggest a novel role of EVI1 as a critical co-factor in a complex that methylates DNA. A question that is not answered here is how EVI1 recognizes the DNA to which it binds in cooperation with a dnDMT. We did not find any significant homology between the published DNA consensus sites for EVI1 and any region of the probe we used, suggesting that either EVI1 recognizes a previously uncharacterized sequence, or recognizes a DNA/chromatin structure rather than a naked, linear bp sequence, or that the cooperation of another protein, in this case Dnmt3b, is necessary to provide a multiprotein surface for recognition and interaction with the DNA. At this time we do not have sufficient data to point at the correct mechanism at the exclusion of the other possibilities listed here. However, our eletrophoretic mobility shift assays show that transfected EVI1 weakly binds to the probe when only endogenous Dnmt3 proteins are present in the extract and that Dnmt3b by itself does not significantly affect the probe migration. In contrast, a band appears when both proteins are strongly co-expressed, suggesting that the DNA recognition and binding could be mediated by combined domains of the two proteins rather than by a single-protein domain.

Since the understanding of the nature of *EVI1* as an oncogene when inappropriately expressed, there has been much emphasis to unravel the tissue-specific pathways that this protein disrupts in a transformed cell. Several years ago, we showed that endogenous *Evi1* is active in undifferentiated murine embryonic stem cells and that it is slowly silenced during the differentiation of the cells *in vitro* until is completely repressed in terminally differentiated cells [50], suggesting a potential role in epigenetic establishment of gene expression patterns during the transition of the cells from a pluripotent phenotype to a lineage-committed state. These older data together with the description of the *Evi1*−/− embryo, the link between EVI1 and DNA methylation, and our data presented in this report strongly support a novel role of EVI1 that is not tissue- or lineage-specific but rather that is actively played in all cells as a dominant participant in *de novo* DNA methylation. It has long been known that abnormal DNA methylation is a hallmark of many cancers [20] though the mechanism of this process is unknown. Based on the data we present here, on our previously published work [17], [48], [49], and on the work by Hoyt et al [3], we would like to propose a novel role of EVI1 as a global player in DNA modification, rather than solely as a lineage- or tissue-specific oncogene, which acts as a co-factor in protein complexes that regulate DNA and chromatin modifications both during normal embryogenesis and in solid and hematopoietic cancers when the gene is inappropriately activated.

## Materials and Methods

### DNA plasmids

The *EVI1* plasmids used in this study have been described [14]. The *EVI1* point mutant *EVI1-(1+6Mut)* contains the H39A and C44A mutations in the first zinc finger and the C190A and C193A mutations in the sixth zinc finger. Murine Dnmt3a fragments were amplified by PCR and cloned with a Flag epitope into the pCMV Vector (Invitrogen/Gibco) using a plasmid encoding Dnmt3a-Myc as template. The latter plasmid and Dnmt3b-Myc are a generous gift of Dr. Chih-Lin Hsien (University of Southern California, Los Angeles). The murine miR-124-3 regulatory region between nt −467 to +28 (where +1 corresponds to the stem-loop start site of miR-124-3) was amplified by genomic PCR and cloned in the promoterless luciferase reporter plasmid pGL4.20 (Promega). All amplified DNA fragments were verified by DNA sequencing.

### Cell infection and transfection

To generate infectious retrovirus particles, we transfected 20 µg of plasmid/10 cm plate in the packaging GP2-293T cells (Clontech) with MegaTrans 1.0 reagent (OriGene). DNA-transfection of adherent cells was performed by the calcium phosphate precipitation method. Murine ES cells were electroporated by using Amaxa Nucleofector II system (Amaxa Biosystems) and were cultured for 10 days in G418 to select those cells in which the plasmid had integrated in the genome.

### Cell culture

293T (ATCC number CRL-11268) and NIH-3T3 (ATCC number CRL-1658) cell lines were maintained as described [51]. Murine ES cells (ATCC number CRL-1934) were maintained in 10 cm gelatin-coated plates in DMEM supplemented with 10% FBS, 2 mM L-Glutamine, 1 mM MEM NEAA, 1 mM Sodium Pyruvate, 500 µM 2-Mercaptoethanol and 10 ng/ml LIF.

### Reporter gene studies

The reporter gene assays were performed with NIH-3T3 cells stably transfected with a plasmid expressing the reporter gene under the control of regulatory regions of miR-124-3. The regions used are: −467 to +28, or −340 to +28, or −109 to +28, where +1 corresponds to the stem-loop start site of miR-124-3. Stably transfected NIH-3T3 cells were transiently co-transfected with effector plasmids. After 72 h, equal numbers of cells were lysed and analyzed. All measurements were done in triplicate.

### Western blot analysis and immunoprecipitation (IP) assays

Cells were harvested 48 h after transfection and treated as described [51]. We used murine mAb M2 to the Flag epitope (Sigma), rat mAb to the HA epitope (Roche), polyclonal rabbit Ab to EVI1 and murine mAb 9B11 to Myc (Cell Signaling Technology), and murine mAbs 64B1446 to Dnmt3a and 52A1018 to Dnmt3b (Imgenex).

### Electrophoretic mobility shift assay (EMSA)

293T cells were transiently transfected with the necessary plasmids and nuclear extracts were used for EMSA. ^32^P end-labeled probes were 147 bp and 252 bp long, corresponding to the −467/−321 and −340/−89 fragments of the miR-124-3 gene. Binding assays were carried out in a 10 µl volume of 10 mM Tris-HCl pH 7.5, 150 mM NaCl, 12.5% glycerol, 5 mM EDTA, 5 mM DTT, and poly-dI:dC (1 µg/reaction). ^32^P end-labeled probes were added after 10 min and incubation was continued for additional 20 min. The samples were loaded on 0.5×Tris-borate-EDTA nondenaturing 4% polyacrylamide gel, electrophoresed at 100 V for 3 h at 4°C, and autoradiographed overnight.

### Chromatin immunoprecipitation (ChIP) assay

ChIP assays were performed with transiently transfected 293T cells by using SimpleChIP™ Enzymatic Chromatin IP Kit (Cell Signaling Technology) according to the manufacturer's instructions. The DNA fragments were analyzed by PCR using two primers (5′-ggagaagtgtgggctcctc-3′ and 5′-aatcaaggtccgctgtgaac-3′) designed to amplify 222-bp of the miR-124-3 regulatory region.

### *In vitro* DNA methyltransferase assays

An 879 bp PCR fragment (−851/+28) derived from miR-124-3 was used as a substrate. EVI1-Dnmt3a/b complexes from ES cells or 293T cells were obtained by immunoprecipitation of the proteins with anti-HA beads. Washed beads (15 µl) were incubated with S-[methyl-^3^H]-adenosylmethionine (Ado-Met) (Perkin Elmer) in presence of the DNA substrate in 50 mM Tris-HCl buffer pH 7.5, 2 mM DTT, 1 mM EDTA, and 0.1% NP-40 in a volume of 30 µl for 24–48 h at 37°C. The incubation mixtures were separated on 6% 0.5×Tris-borate-EDTA nondenaturing polyacrylamide gel, transferred onto a positively charged nylon membrane (Roche), and exposed to a film for 2 weeks.
